# Malignant transformation of a solitary tracheal papilloma in the absence of human papillomavirus infection in a patient with 48, XYYY syndrome: case report

**DOI:** 10.1186/s13019-023-02275-5

**Published:** 2023-04-17

**Authors:** Awrad Nasralla, Esther Lau, Ranjan Sur, Yaron Shargall

**Affiliations:** 1 Department of General Surgery, University of Alberta, Edmonton, AB Canada; 2 Division of Thoracic Surgery, McMaster University, Hamilton, ON Canada; 3 Department of Radiation Oncology, Juravinski Cancer Centre, McMaster University, Hamilton, ON Canada

**Keywords:** Trachea, Papilloma, Carcinoma

## Abstract

The diagnosis and management of tracheobronchial papilloma is challenging due to its rarity, and non-specific presenting symptoms. Small percentage undergoes malignant transformation. Herein, we report an unusual case of tracheal papilloma initially misdiagnosed as chronic obstructive pulmonary disease (COPD) in 36-year-old male with triple Y syndrome. It was successfully treated with local debridement and brachytherapy. To the best of our knowledge, this is the first description of brachytherapy for such a condition.

## Introduction

Papilloma is a benign neoplasm, most commonly occur in the genitalia or the oral cavity. Usually in relation to human papilloma virus (HPV) infection. It rarely affects the airways, predominantly involve the larynx, however, involvement of the trachea has been reported to be around 5% of the cases [1, 2]. Triple Y syndrome is a very rare condition, and it is phenotypically similar to Klinefelter syndrome. Patients with such syndrome are prone to respiratory infections, and asthma [3]. Likely the history of heavy smoking besides the predisposition to respiratory disease delayed his diagnosis and treatment. This patient had squamous papillary lesion with focal changes of carcinoma in situ without HPV infection. It was treated with debridement and adjuvant brachytherapy.

### Case presentation

**Fig. 1:**
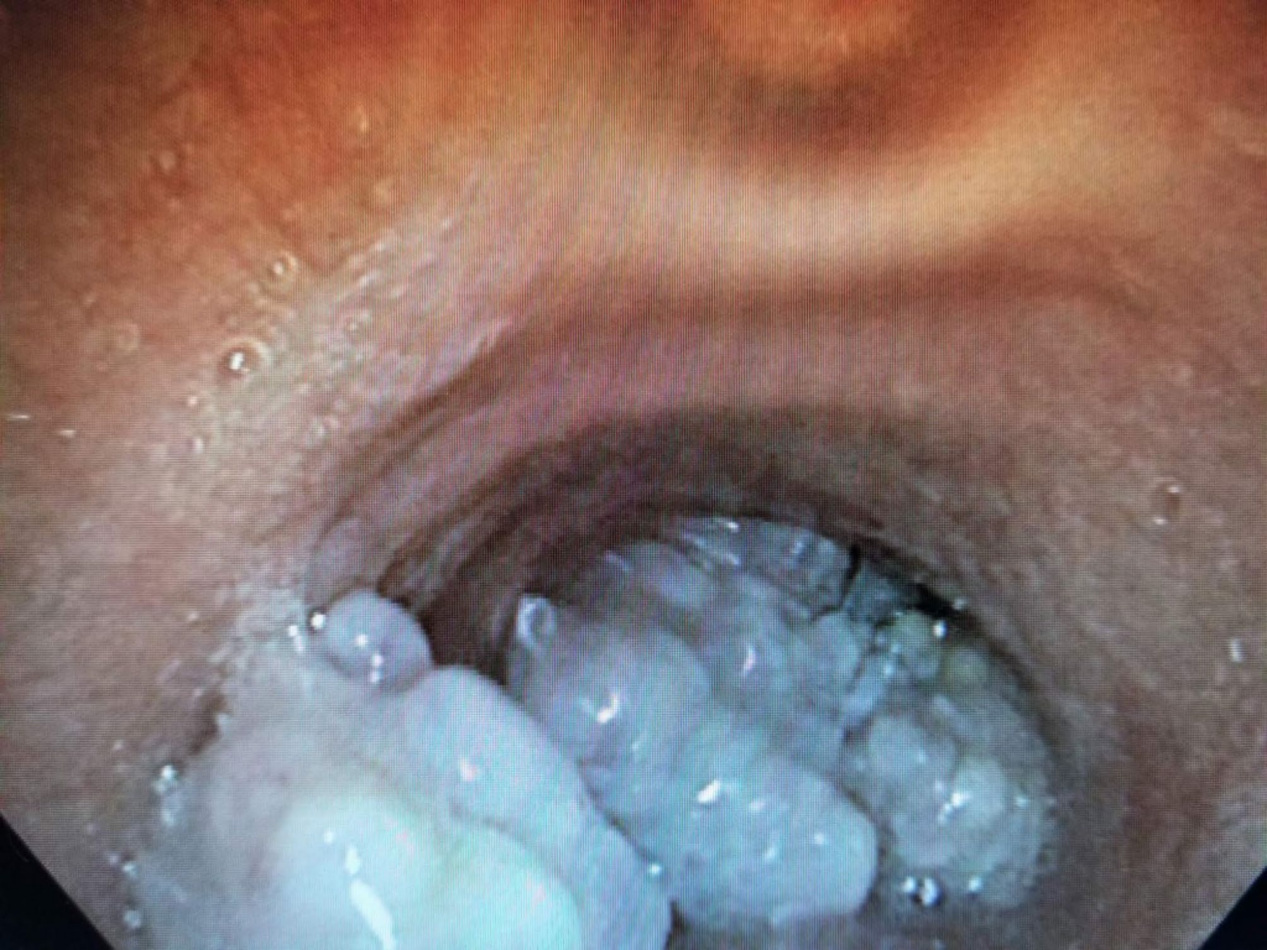
Bronchoscopic appearance of the tracheal masses (cauliflower like lesions)

**Fig. 2:**
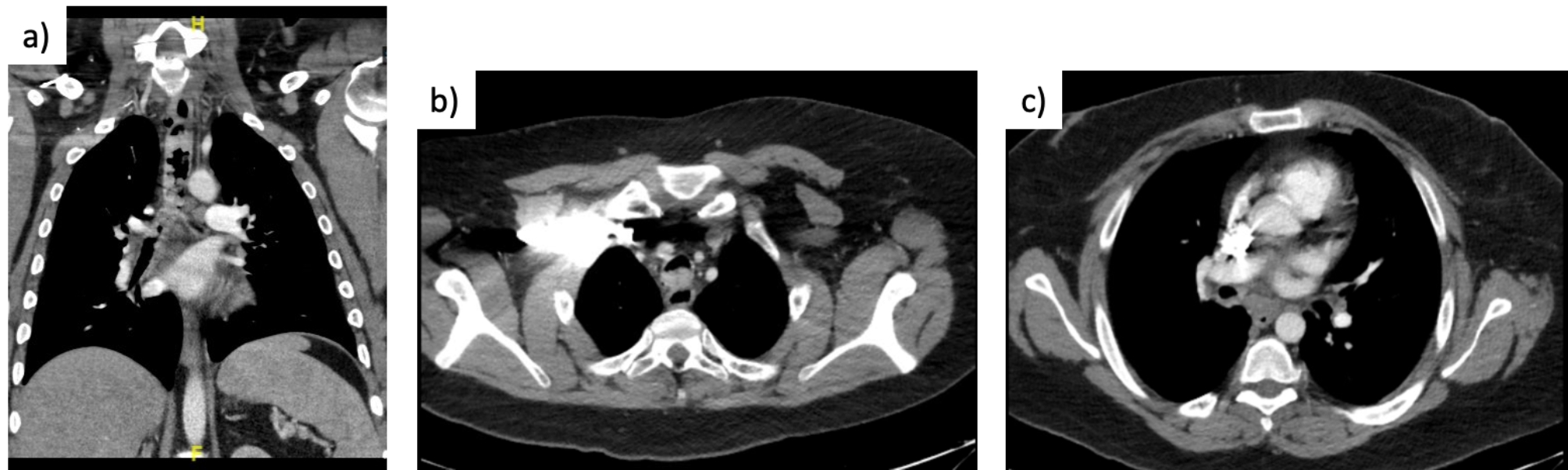
CT scan demonstrating lesions along almost entire length of trachea (a), causing near complete occlusion in proximal trachea (b) and right mainstem bronchus (c)

A 36-year-old male with 48, XYYY karyotype and 15-pack-year smoking history presented with 2-year history of progressive dry cough, raspy breathing, and dyspnea. His past medical history include developmental delay, attention-deficit/hyperactivity disorder (ADHD), gastroesophageal reflux disease (GERD), behavior disorder (aggressiveness, and self-harming), and obesity. He was initially treated as a case of chronic obstructive pulmonary disease (COPD), however, he did not improve, as such further investigations were carried out. He underwent bronchoscopy, which revealed irregular, lobulated tissue starting from the fourth tracheal ring throughout the entire trachea, extending into the bronchus intermedius with near total obstruction (Fig. 1). Chest CT showed diffuse endotracheal lesions involving the mid-distal tracheal mucosa, extending into the right mainstem bronchus and bronchus intermedius, with 80% occlusion of the trachea (Fig. 2). No endobronchial disease within the left mainstem, lobar or segmental bronchi. No suspicious pulmonary nodule or mass otherwise.

The patient underwent rigid bronchoscopy and partial tumor debridement. When the patient was still breathing spontaneously, he was intubated with a laryngeal mask initially and a full flexible video-assisted bronchoscopy was performed. Several biopsies were taken using biopsy forceps and subsequently the laryngeal mask was pulled out and a rigid bronchoscopy was performed. The airway was intubated, and debridement of the tumor was done using biopsy forceps under direct vision with the flexible bronchoscope within the rigid scope. During this part of the procedure, he was ventilated with jet ventilation.Then, he became hypoxemic and eventually arrested. He required two minutes CPR with an immediate intubation with a single lumen endotracheal tube. He developed bilateral pneumothorax, for which chest tube was inserted on each side. The patient was transferred to the Intensive Care Unit (ICU) intubated and sedated with no inotropic support. A bronchoscopy was repeated which showed one residual area of a small papilloma in the mid trachea, originating from the membranous part. The rest of airways were clear and patent. He was extubated, transferred to the ward in a stable condition, then discharged home few days later.

Histopathological examination revealed squamous papillary lesion with focal carcinoma in situ and p16 immunohistochemistry negative. He was thereafter referred to radiation oncology and underwent brachytherapy (two fractions of 700 cGy) with a good clinical and endoscopic response. He remains well 6 months after treatment, no recurrence.

## Discussion

This is an unusual case for several reasons: presentation with solitary tracheobronchial papilloma which is rare compared to papillomatosis of upper respiratory tract; the existence of malignant transformation which is uncommon at 3–7% of patients ​ [4, 5] and even more unusual is that HPV infection was not implicated in this case. This patient has a history of Triple Y syndrome which has fewer than ten cases described in literature. Those with such syndrome may experience learning difficulties, speech delay and difficulties, and emotional instability. There is no known association between Triple Y syndrome and respiratory papillomatosis or any type of cancer, but asthma is often a clinical feature and may have obscured the eventual diagnosis [3]. Several theories have been proposed about the potential causes for malignant transformation of respiratory papillomatosis, such as smoking, HPV infection, and localized trauma [6]. Theories of malignant transformation include synergistic effect of smoking and HPV infection, abnormal expression of genes such as tumor suppression gene [7] .

The clinical presentation is variable which make the diagnosis more challenging. Bronchoscopy could be both diagnostic and therapeutic in some cases. Respiratory papillomatosis is primarily treated by resection surgical or endoscopic. Other treatment modalities include laser therapy, interferon α, external beam radiation or photodynamic therapy [8]. The goal of the treatment is to remove the tumor and to maintain a patent airway. The choice between these depends on the location, extent, presence or absence of HPV infection, and the expertise available.

Because of the location of the papilloma in the airway, the team should be prepared in case inadvertent events happened such as pneumothorax that likely occurred during the jet ventilation during the case. It is essential to discuss the case and the operative procedure with the anesthesia team prior to surgery to plan the airway management intraoperatively.

Given the rarity of the tracheal papillomatosis, most of the information were based on case reports. Local recurrence might happen, as such close follow up is important. However, there are no guidelines regarding the surveillance post resection, further studies are needed.

## Conclusion

Primary tracheal papilloma is rare. Usually, patients present with non- specific respiratory symptoms, as such high clinical suspicion is needed. We recommend CT scan and bronchoscopy to establish the diagnosis. We report a successful treatment with local therapy using endoscopic debridement followed by brachytherapy. The main treatment is resection, however, the rule of adjuvant therapy needed to be studied further.

## Data Availability

Provided in the case report.

## Abbreviations

CT: Computed Tomography
HPV: Human Papilloma Virus
